# Ex vivo biomechanical evaluation of extracapsular stabilization with quasi-isometric points in canine cranial cruciate ligament-deficient stifles

**DOI:** 10.1186/s12917-023-03656-7

**Published:** 2023-07-24

**Authors:** Wei-Ru Hsu, Cheng-Chung Lin, Cheng-Yuan Sun, Ching-Ho Wu

**Affiliations:** 1 Institute of Veterinary Clinical Science, School of Veterinary Medicine, National Taiwan University, No. 153, Sec. 3, Keelung Rd., Da’an Dist, Taipei City, 106328 Taiwan (R.O.C.); 2 Department of Electrical Engineering, Fu Jen Catholic University, New Taipei City, Taiwan (R.O.C.)

**Keywords:** Extracapsular stabilization, Cranial cruciate ligament deficiency, Isometric point, Stifle kinematics, Joint stability

## Abstract

**Background:**

Cranial cruciate ligament (CCL) disease is one of the most common causes of lameness in dogs. The extracapsular stabilization (ECS) utilizing bone anchors and monofilament nylon leader was an alternative treatment for CCL-deficient (CCLD) dogs. However, the biomechanical response of the canine stifle to such a surgical repair strategy in conjunction with the use of recently reported quasi-isometric anchoring points remains unclear. The objectives of the study were to evaluate the mobility and stability of CCL-intact, CCLD, and CCLD stifles repaired with ECS at two different pairs of quasi-isometric points (quasi-IPs).

**Methods:**

Twelve stifle specimens from 7 dogs underwent mobility and stability tests under 4 different conditions, namely, CCL-intact, CCLD, and ECS-repaired at 2 different pairs of quasi-IPs (referred to as ECS-IP1 and ECS-IP2). The mobility tests evaluated 6 degrees-of-freedom stifle kinematics during flexion and extension. The stability tests involved cranial drawer and tibial internal rotation (IR) tests at various stifle opening angles and quantifying the cranial tibial translation (CTT) and tibial IR angles under constantly applied loadings.

**Results:**

The ECS repaired at quasi-IPs was shown to restore cranial instability of the stifles with averaged CTT magnitudes < 1.4 mm. During the tibial IR test, the ECS treatments resulted in significantly less tibial IR compared to those in intact CCL stifles. The mobility tests showed similar results.

**Conclusion:**

The 2 chosen pairs of quasi-IPs were shown to effectively correct the excessive CTT caused by CCLD stifles, whereas the excessive tibial external rotation in comparison to those of intact stifles should be considered for its subsequent influence on joint alignment and the contact pressure applied to the stifle joint.

## Introduction

Cranial cruciate ligament (CCL) disease is one of the most common causes of lameness in dogs [1–4]. Common surgical treatments of CCL disease include intracapsular reconstruction, extracapsular stabilization (ECS), and corrective tibial osteotomy [5]. Some recent evidence showed that the corrective tibial osteotomy procedures, particularly tibial plateau leveling osteotomy, showed better postoperative outcomes than other treatments in terms of functional recovery and halting OA progression [6–10]. In contrast, while ECS procedures were shown to yield comparable or inferior postoperative outcomes [9–11], they remained common surgical options for CCL diseases due to the lower costs and technical demands [1, 3, 12]. The ECS procedures were also advantageous to enable the provision of immediate internal rotation (IR) stability [13].

Numerous ECS procedures had been proposed including lateral fabellar suture (LFS), TightRope, bone anchor techniques, etc. [14–17]. Among the approaches, the LFS and TightRope had been widely investigated for their postoperative biomechanical influences on the stifle joint [1, 2, 18, 19]. However, the LFS that utilized the circumfabellar sutures as the femoral anchoring point was found to be less isometric during the stifle range of motion [20]. Anisometry of extracapsular sutures resulting in variations in prosthesis tension throughout the range of stifle motion can increase the risk of postoperative complications [21]. Moreover, the shortened lifespan of the ECS may lead to premature failure before periarticular fibrosis develops, thereby potentially compromising the long-term outcome. Most quasi-isometric point pairs on the stifle have been assessed for over a decade [1, 12, 18, 20, 22–24], but there are controversial results, possibly due to inconsistent evaluation approaches, prosthetic materials, and surgical techniques used. Nonetheless, in recent years, the points distal to the lateral femoral fabella, near the insertion of the patella tendon at the tibial crest, and at the tubercle caudal to the long digital extensor groove were the most recognized potential quasi-isometric points in ex vivo and in vivo gait studies [1, 12, 18–20, 23].

Some modifications to the ECS, such as the use of bone anchors and tunnels, were proposed in an attempt to allow more accurate placement of anchor sites to achieve better isometry of the suture [25] and prevent possible complications associated with circumfabellar prostheses, such as tearing or loosening of the femoral-fabellar ligament. The TightRope procedure, taking the point craniodistal to the lateral fabella–femoral condyle junction as the femoral anchoring point [16], may ensure a better isometry of the suture, but the use of the braided multifilament suture increases the incidence of infection [26].

In an attempt to overcome the limitations associated with circumfabellar implants and multifilament sutures, the technique utilizing bone anchors and monofilament nylon leader (MNL) was an alternative treatment for CCL-deficient (CCLD) dogs [27]. However, to the author’s knowledge, while such a surgical repair strategy had been used in clinics, the effects of the treatment in conjunction with the use of recently reported quasi-isometric anchoring points on stifle stability and mobility remain unclear.

The objectives of the study were to evaluate the stability and 6 degrees-of-freedom mobility of cranial cruciate ligament (CCL)-intact and CCLD stifles and of CCLD stifles repaired with ECS utilizing bone anchors and MNL sutures at two different pairs of quasi-isometric points (IPs). To this end, a custom-made biomechanical testing platform in conjunction with an optical motion capture system was built for the ex vivo biomechanical evaluation of the stifle joint.

## Results

### Specimen collection and preparation

The 12 recruited pelvic limbs were obtained from 7 adult canine cadavers. The mean body weight was 17.97 ± 2.11 kg (median: 17.2 kg; range: 16.5–22.6 kg), with ages ranging from 1 to 14 years old. Breeds included mongrel dogs (5/7), Border Collie (1/7), and Pit bull (1/7). Four-sevenths of the group were female, and the others were male.

### Kinematic deviations in mobility tests

**Fig. 1:**
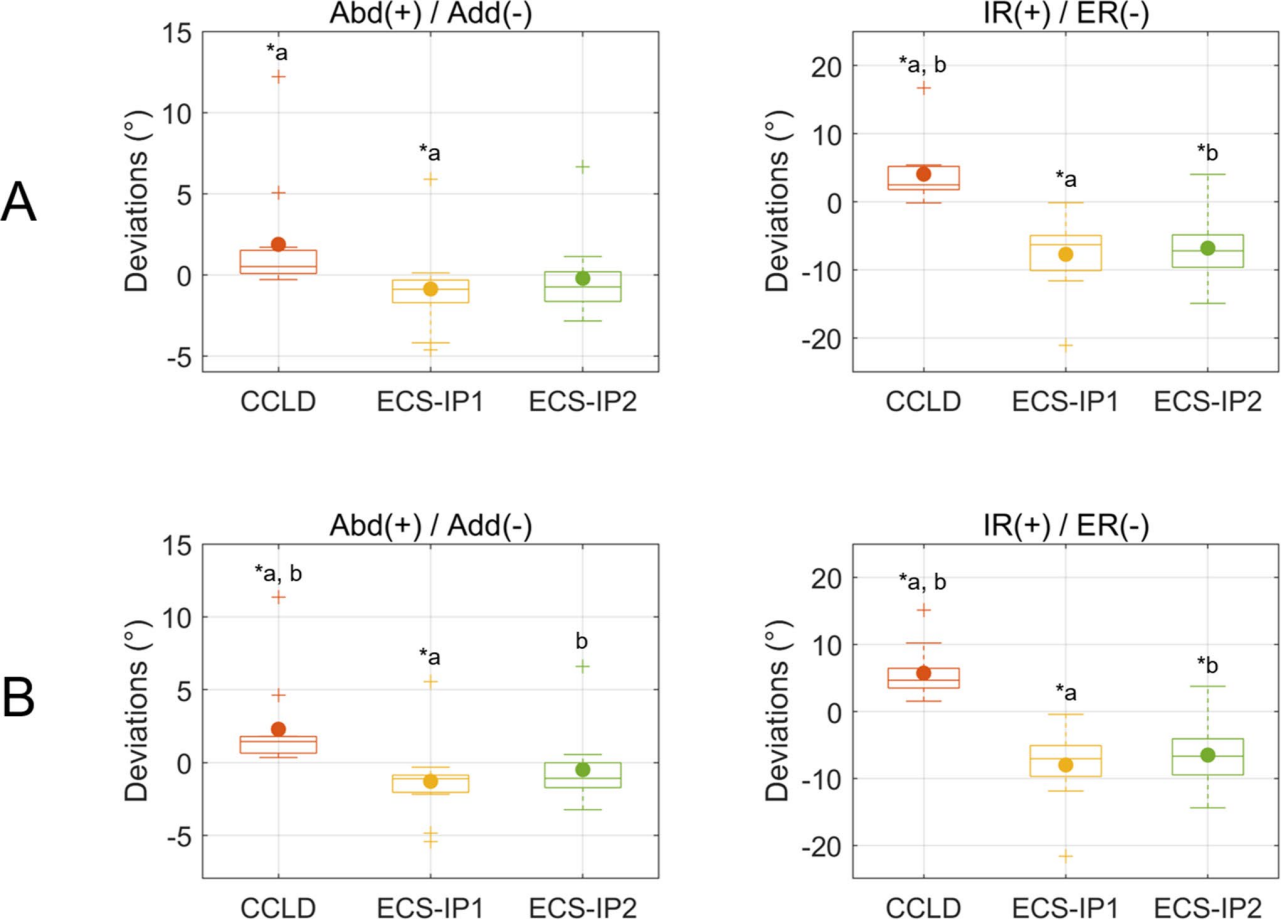
The boxplots show the deviations of abduction/adduction (Abd/Add) and internal/external rotation (IR/ER) of the CCLD and ECS groups compared to the CCL-intact stifles during (**A**) stifle extension (i.e., quadriceps with pulling force) and (**B**) flexion (i.e., quadriceps without pulling force). The central lines of the boxes and solid dots represent the median and mean values, respectively; the edges of the boxes represent the first and third quartiles of the distributions; the cross marks signify the outliers; and the ranges of the whiskers indicate the upper and lower extremes. Asterisks indicate significant kinematic deviations. Values with the same letters (a-b) differ significantly (*p* < 0.05)

**Fig. 2:**
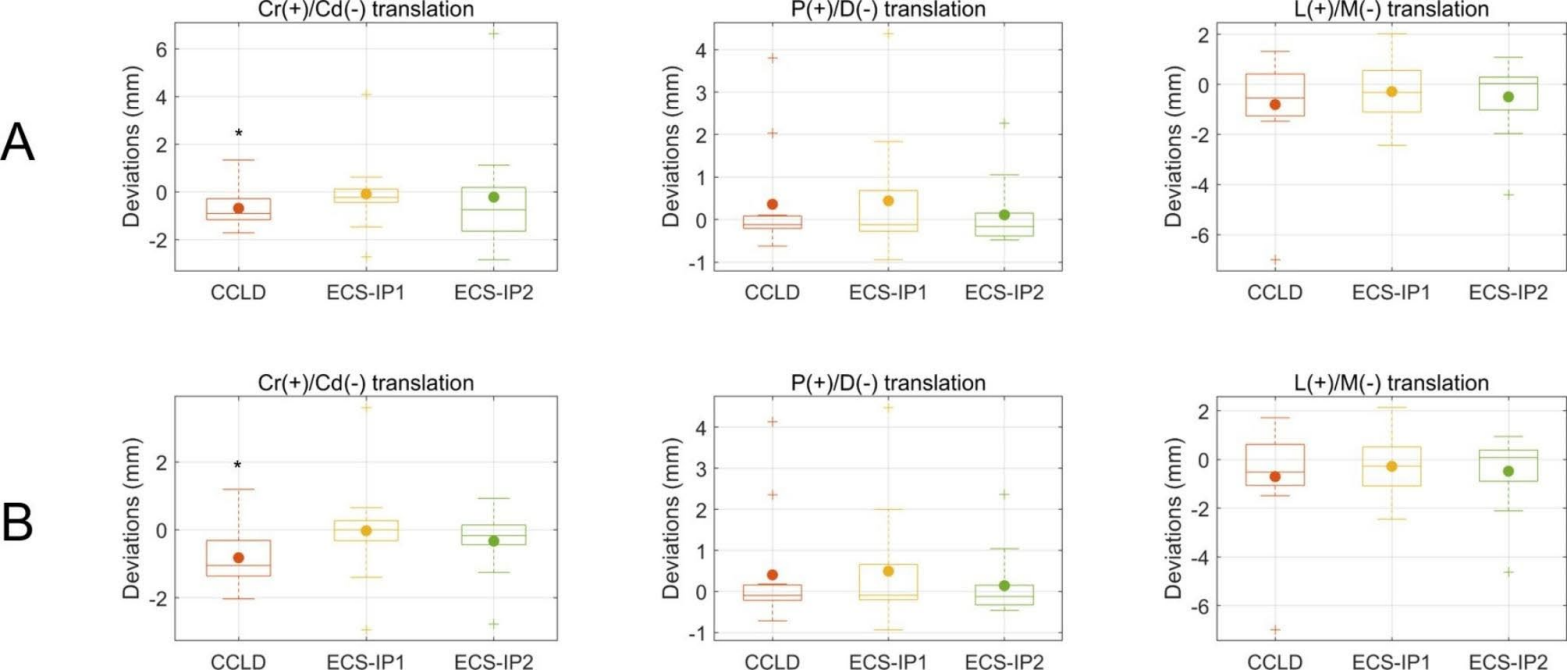
The boxplots show the deviations of stifle joint center translations in cranial/caudal (Cr/Cd), proximal/distal (P/D), and lateral/medial (L/M) translations of the CCLD and ECS groups compared to the CCL-intact stifles during (**A**) stifle extension (i.e., quadriceps with pulling force) and (**B**) flexion (i.e., quadriceps without pulling force). The central lines of the boxes and solid dots represent the median and mean values, respectively; the edges of the boxes represent the first and third quartiles of the distributions; the cross marks signify the outliers; and the ranges of the whiskers indicate the upper and lower extremes. Asterisks indicate significant kinematic deviations

During the stifle mobility test, significantly higher coupled abduction (Abd) and IR were observed in CCLD stifles than in CCL-intact stifles, with median deviations less than 1.4° and 4.7°, respectively (Fig. 1). The ECS groups showed opposite motion patterns, in which ECS-IP1 resulted in significantly coupled adduction (Add) with median deviations less than 1.1°. Both ECS groups resulted in significantly coupled external rotation (ER) compared to the CCL-intact stifles, with median deviations ranging from 6.3° to 7.2° (Fig. 1). For stifle joint center translations, only the CCLD stifle showed significantly deviated joint translation in the cranial/caudal (Cr/Cd) direction (Fig. 2).

### Joint stability in the cranial drawer test

**Fig. 3:**
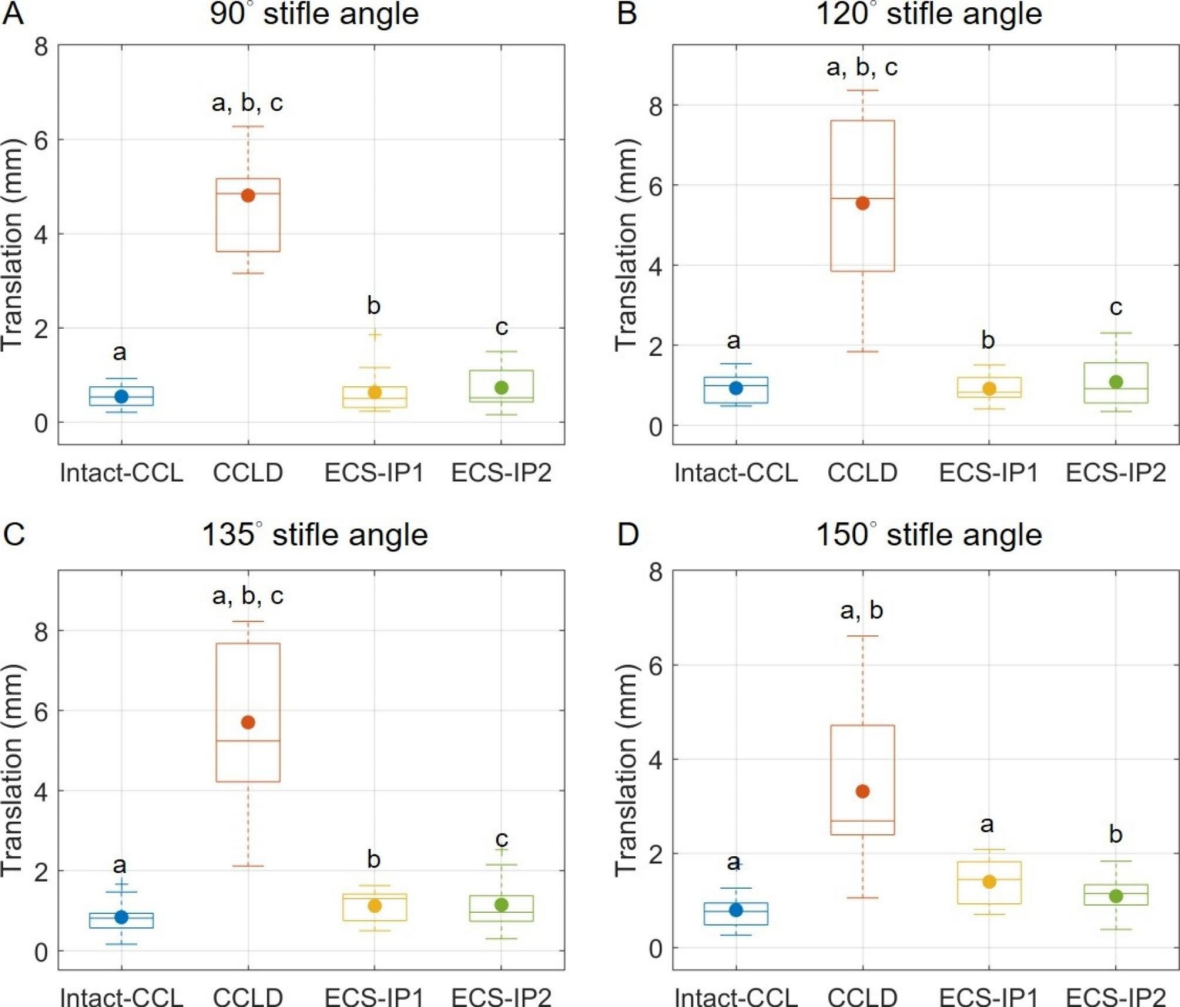
The boxplots show the distributions of cranial tibial translations for the intact CCL, CCLD, and ECS groups in cranial drawer tests. The central lines and solid dots in the boxes represent the median and mean values, respectively; the edges of the boxes represent the first and third quartiles of the distributions; the cross marks represent outliers; and the ranges of the whiskers indicate the upper and lower extremes. Values with the same letters (a-c) differ significantly (*p* < 0.05)

The averaged cranial tibial translation (CTT) of the CCLD stifles ranging from 3.3 to 5.7 mm were significantly greater than those in other CCL statuses (Fig. 3). For stifle opening angles lower than 135°, no significant differences were detected among the intact CCL and both ECS groups, where the averaged CTT values were all below 1.2 mm. At a stifle opening angle of 150°, ECS-IP1 had a significantly higher CTT value than that in the intact CCL stifle, with a mean difference of 0.6 mm.

### Joint stability in the tibial internal rotation test

**Fig. 4:**
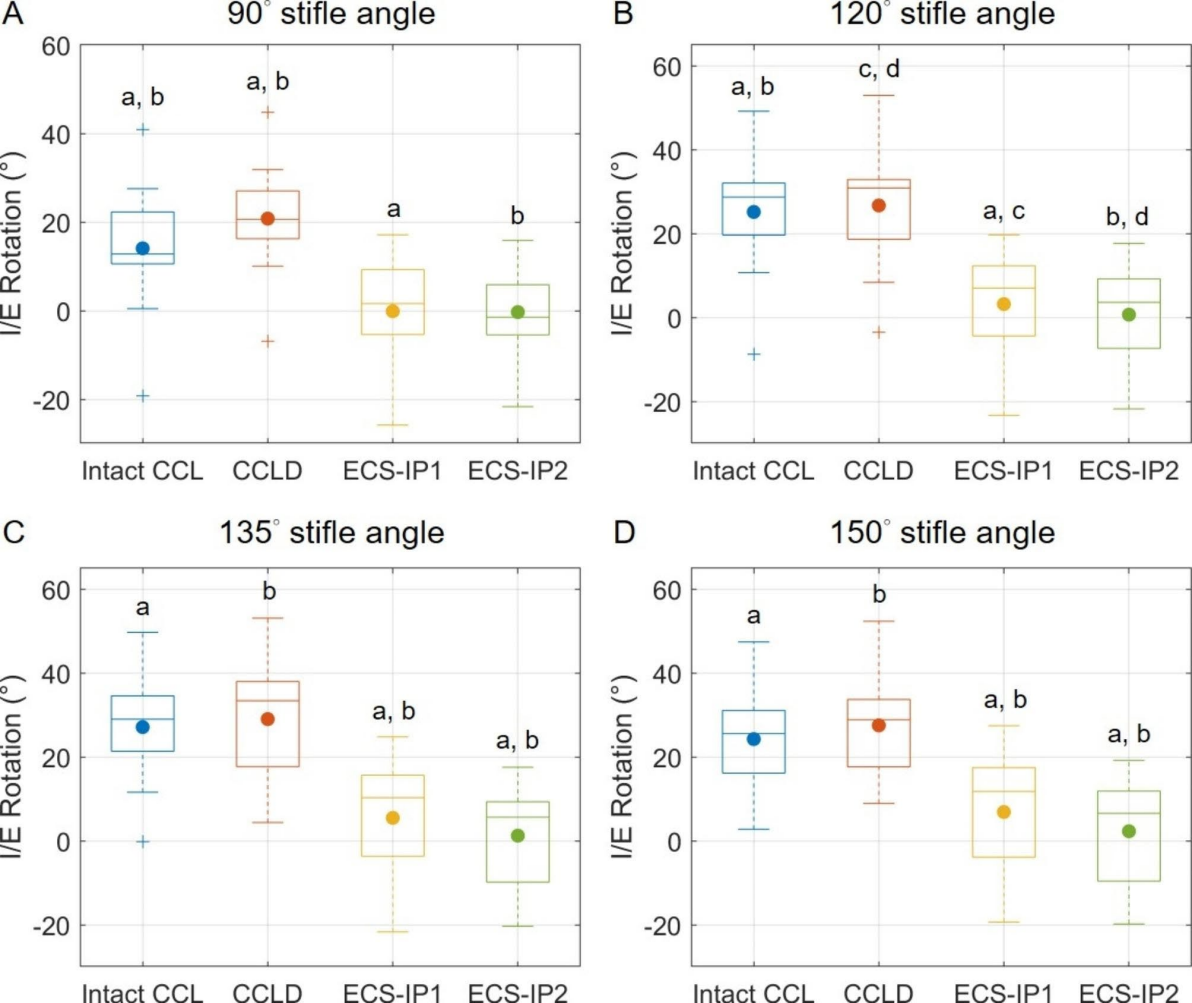
The boxplots show the distributions of maximal tibial internal rotation angles for the intact CCL, CCLD and ECS groups in tibial internal rotation tests. The central lines and solid dots in the boxes represent the median and mean values, respectively; the edges of the boxes represent the first and third quartiles of the distributions; the cross marks represent outliers; and the ranges of the whiskers indicate the upper and lower extremes. Values with the same letters (a-d) differ significantly (*p* < 0.05)

In general, the averaged tibial IR angles ranging from 20.8° to 29.1° in the CCLD stifle were the highest among the groups and were significantly greater than those in the CCL-intact stifle at the 90° stifle opening angle and those in the ECS groups (Fig. 4). Significantly diminished tibial IR was found in both ECS groups when compared with those of CCL-intact and CCLD groups. The means of the IR angles were within 6.9°. At stifle opening angles of 135° and 150°, ECS-IP2 restricted the tibial IR angles significantly more than ECS-IP1.

## Discussion

The present study assessed the stability and mobility of CCL-intact and CCLD stifles and CCLD stifles repaired with ECS using a custom-made biomechanical testing platform in conjunction with a motion capture system. To the author’s knowledge, the kinematic response of a canine stifle repaired with ECS utilizing bone anchors and MNL sutures at pairs of quasi-IPs has never been documented. The results demonstrated that there were no significant differences in the kinematics between ECS treatments with different quasi-IPs during F/E. Transecting the CCL yielded a significantly higher CTT, and the ECS treatments effectively repaired the cranial instability of the stifle with CTT magnitudes close to those in CCL-intact stifles. However, compared to the intact CCL stifles, others showed overrestricted tibial IR after surgery, especially in ECS-IP2.

Several preceding studies have revealed notable cranial tibial subluxation after CCLD progression [1–3, 24, 28–30], but only a few studies have assessed and compared the CTT between different pairs of quasi-IPs [19, 24, 31, 32]. It is expected that suturing at different positions affects the resistance capability in tibial cranial translation, as sutures with alignment closer to the craniocaudal direction can contribute a greater tensile force component to counteract tibial cranial translational forces [12]. However, only a few studies reported that suturing at T1 was found to be more effective in resisting CTT than suturing at T2 or T3 [19, 24]. No significant differences in CTT between ECS-IP1 (i.e., T1) and ECS-IP2 (i.e., T3) were found in the present study during both mobility and stability tests (Figs. 1 and 2). This contradiction to the expectation may be attributed to the method of securing joint stability. The assurance of the negative manual cranial drawer (CD) test does not represent consistent suture tensions among ECS procedures and subjects owing to the different alignments of suture lines at the two IPs and the existence of intraoperator variability.

While the ECS groups appeared to effectively restore the stifle translation characteristics during stifle F/E (Fig. 2), significantly coupled external rotation was also found when compared with the CCL-intact and CCLD stifles (Fig. 1). This finding was in agreement with several previous reports [19, 33, 34]. Aulakha et al. evaluated the 3D femorotibial translational and rotational movements for ECS at two tibial attachment sites via a weight-bearing model and found excessive tibial external rotation at various stifle flexion angles [19]. However, the higher external rotation, as a result of the longer lever arm of a more cranial suture anchor point [19], was not observed in the current study (Fig. 1). This discrepancy may arise topically from different experimental scenarios and inconsistent tensile forces of the sutures. In the present study, stifle F/E was created by controlling quadriceps forces without sustained weight-bearing, which should lead to notable differences in compressive forces in the stifle joint. The differences in the testing scenarios may thus lead to variations in the kinematic responses to the surgical treatments. While the suture tensions were not measured, we speculated that the suture lines anchoring at the more cranial position in the tibia may lead to a lower tensile force owing to a slightly flatter orientation of the suture line and the procedure of securing the sutures, compromising the resulting axial torque.

Furthermore, in a recent study by Del Carpio et al., the ECS-stabilized stifle remained significantly less externally rotated than the intact stifle, contrary to the findings of most previous studies and the present study [35]. They suggested that the ECS can lead to normal stifle kinematics with adequate suture tensioning. It thus appeared that with a similar surgical technique, inconsistent suture tensioning plays an important role in different postoperative stifle kinematics. A previous study also indicated that there is variability in the tension applied during ECS application, both within and between surgeons, which may lead to discrepancies in clinical outcomes [36]. Further studies are warranted to quantify the suture tension and assess the impact of different suture tensions.

The present study utilized an IR torque of 0.6 Nm, which was smaller than that in preceding studies [34, 37], during the tibial IR test. The reason for this choice was twofold. First, we intended to reproduce the tibial IR angles close to those during daily ambulation [38, 39] as the resultant torque applied may be a better representative of those applied on the stifle joint during a dog’s gait. Second, our preliminary tests using a 2 Nm torque on the native joint repeatedly yielded gradually increasing tibial IR angles, which may indicate that the CCL or other surrounding tissues were partially ruptured. Since there were repeated measurements on a specimen in our experimental protocol, any tissue damage during the experiment should be avoided. While the current IR torque appeared to be incapable of distinguishing the stability between the intact and CCLD groups, as shown in preceding reports [1, 40], the overconstraints on IR after ECS were clearly revealed. In addition, while the data were not presented, excessive tibial external rotations in comparison to the intact stifle were observed in both ECS groups before the legs were loaded. This may indicate that to repair the cranial instability of CCLD stifles with the ECS approach, the native alignment of the joint was also changed. Such restriction on the physiological internal rotation may increase the loads exerted on the sutures implanted, affecting the duration of the ECS.

In the present study, we utilized a marker-based motion capture system to obtain precise measurements of 3D segmental kinematics during testing. To eliminate the influence of soft tissue artifacts that can affect kinematic measurements [41], we rigidly attached marker clusters to the diaphysis of bones rather than skin surfaces. The motion capture system employed in the study is commercially available and has been proven to be highly precise, with sub-millimeter accuracy [42]. With proper camera configuration and system calibration, systematic errors of the motion capture system on the stifle kinematics measurement are expected to be insignificant.

Some limitations regarding our experimental design should be noted. First, the current study employed repeated measurements on a sample for four stifle statuses, which inevitably led to potential errors resulting from the mechanical changes in the degraded tissues even though we randomized the order of different tests. Second, the rates of pulling or releasing the quadriceps muscle nylon lines were not strictly controlled, which may have resulted in slight between-trial variations in the created stifle kinematics. Third, the lack of precise control of the tension of suture materials may have increased the between-subject variability in the stability measurements and the stifle kinematics after ECS.

## Conclusions

In conclusion, ECS using bone anchors and MNL sutures attached at both pairs of IPs led to similar stifle kinematics at the stifle, effectively diminishing the tibial cranial motion caused by CCLD stifles and repairing the cranial stability of the stifle joint. However, the overconstrained tibial IR after ECS in comparison to that of the intact stifle was observed during both the mobility and stability tests. It appeared that while the ECS at the quasi-IPs enabled the restoration of the cranial stability and mobility of the stifle, the subsequent biomechanical responses owing to the overconstrained IR and changes in the joint alignment should be considered.

## Methods

### Specimen preparation

Twelve cadaveric pelvic limbs (6 left and 6 right) were harvested from 7 client-owned adult dogs that were euthanized or that died of reasons unrelated to the study. The included dogs were medium- to large-breed dogs with body weights between 15 and 30 kg (body condition score within 4–6/9) and free from musculoskeletal abnormalities at the stifle joints. Since there were no laboratory dogs used for the study, the breed selection was not restricted and the range of the body weights for enrolled dogs was defined considering the majority of CCLD patients in our hospital. The owners provided written informed consent for the data collection, and the study protocol was approved by Institutional Animal Care and Use Committee of National Taiwan University (IACUC number: NTU-109-EL-00070). All confirm that all methods used in the study were performed in accordance with the relevant guidelines and regulations.

The pelvic limbs were disarticulated from the coxofemoral joints. All muscles were stripped off except for an approximately 5-cm-long quadriceps muscle connected to the patella. The periarticular soft tissues at the stifle were preserved. The specimens were stored at -20 °C and thawed at 4 °C for 24 h before preparation and testing. Soft tissues were kept moist by spraying 0.9% saline solution on the tissues repeatedly throughout the specimen preparation period and experiment.

### Equipment setup

**Fig. 5:**
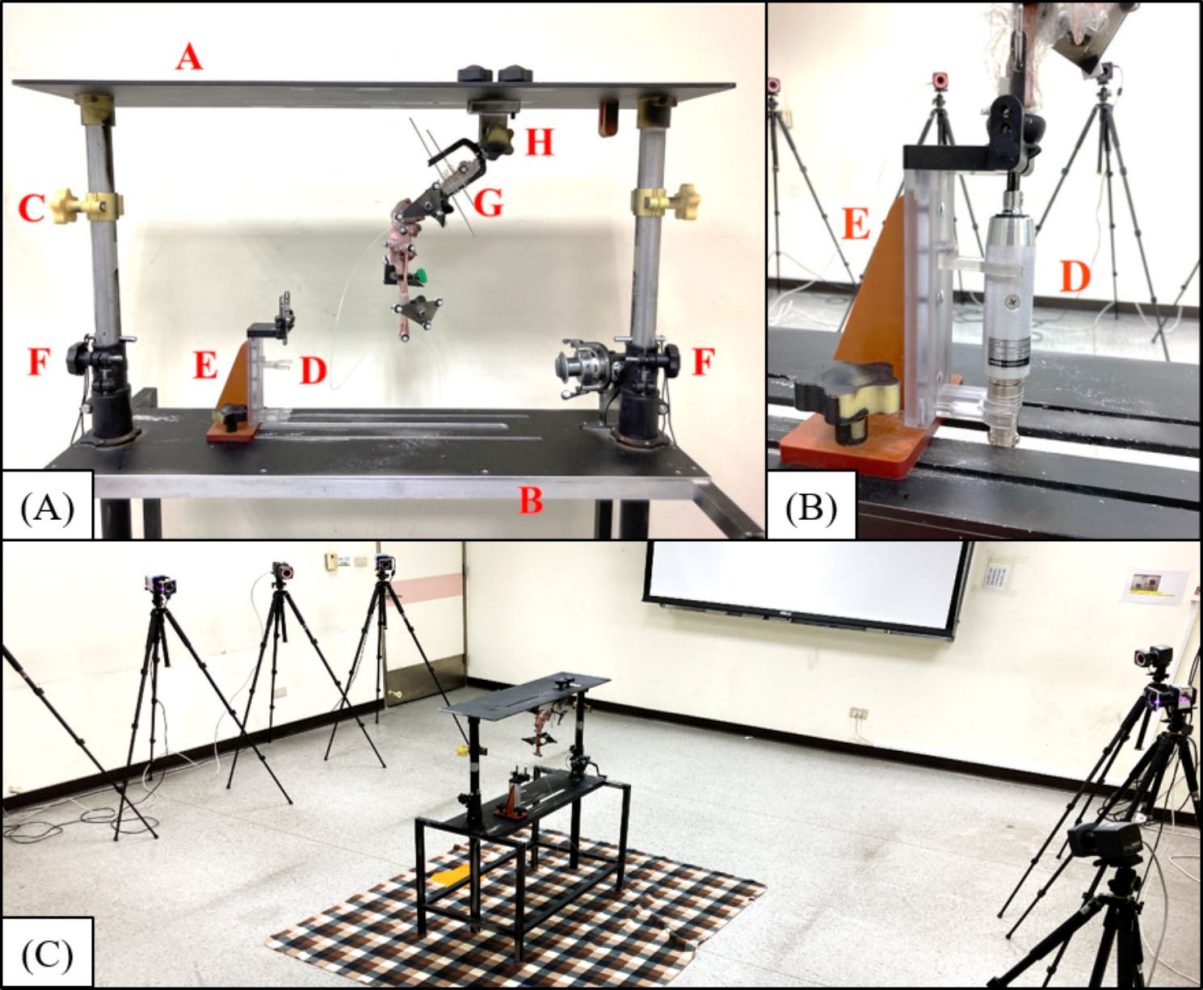
(**A**) Custom-made biomechanical testing platform. Each component is described below. A: an adjustable top plate; B: a base plate with skid rails; C: a height-adjustable groove to guide the suture providing the cranial force; D: a torsionmeter; E: a sliding base on the base plate; F: fixation screws used for adjusting the height of the top plate; G: a metal fixture used for stabilization of the distal femur; and H: a bolt used for adjustment and fixation of the femoral angle. (**B**) The torsionmeter assembly used for the tibial internal rotation test. (**C**) The setup of infrared cameras and the testing platform in the laboratory space

A custom-made biomechanical testing platform was built to facilitate the mobility and stability tests of the stifle joint (Fig. 5A). The top plate of the platform was adjusted by two vertical retractable columns to accommodate various limb lengths and stifle angles. The proximal femur was affixed to a metal fixture by two 2.5-mm Steinmann pins, which were attached to the top plate with a bolt. A sliding base connected to a torsionmeter on the base plate held the distal end of the tibia (Fig. 5B), which provided stabilization during tests and allowed torque measurement during internal/external rotation of the stifle joint.

An optical motion capture system equipped with 9 infrared cameras (6 Bonita 10 cameras and 3 Vero v2.2 cameras, VICON, Oxford Metrics, UK) was set up surrounding the testing platform for measuring the stifle kinematics (Fig. 5C). Retroreflective markers (7.9-mm diameter pearl markers with a 1/2’’ flexible base, B & L Engineering Inc., Santa Ana, CA, USA) were categorized into anatomical markers and tracking markers. Anatomical markers glued on drawing pins were directly attached to bony landmarks, namely, the greater trochanter, bilateral femoral condyle, proximal and distal tibial crests, fibular head, and bilateral malleolus, which were then used to determine the anatomical frame of the femur and tibia. The tracking markers, which were used for tracking skeletal motion during tests, were attached to the 3D-printed marker plates. Overall, the diaphysis of the femur and tibia were each equipped with two marker plates by means of hose clamps.

### Testing tasks

An anatomical calibration was carried out in each limb specimen positioned at a 135° stifle opening angle to establish their own anatomical frame of the femur and tibia. The tracking marker arrays expressed in the corresponding anatomical frame were taken as the “marker templates” used for dynamic motion tracking. After completion of the calibration, the anatomical markers were removed as they impeded the subsequent surgical procedure, and the limb specimens with intact CCL underwent the mobility and stability tests as described below.

The mobility tests aimed to assess the 6 degrees-of-freedom kinematics of the stifle during the full range of flexion and extension. During the tests, each specimen was first secured at the metal fixture on the top plate of the testing platform with the femoral long axis parallel to the floor. The mobility tests were further divided into two conditions based on whether there was a pulling force on the quadriceps muscle: (1) quadriceps with pulling force, which was carried out by manually applying forces on the quadriceps muscle to mimic an extension activity and (2) quadriceps without pulling force, which reproduced the passive stifle flexion from full extension driven by gravity.

**Fig. 6:**
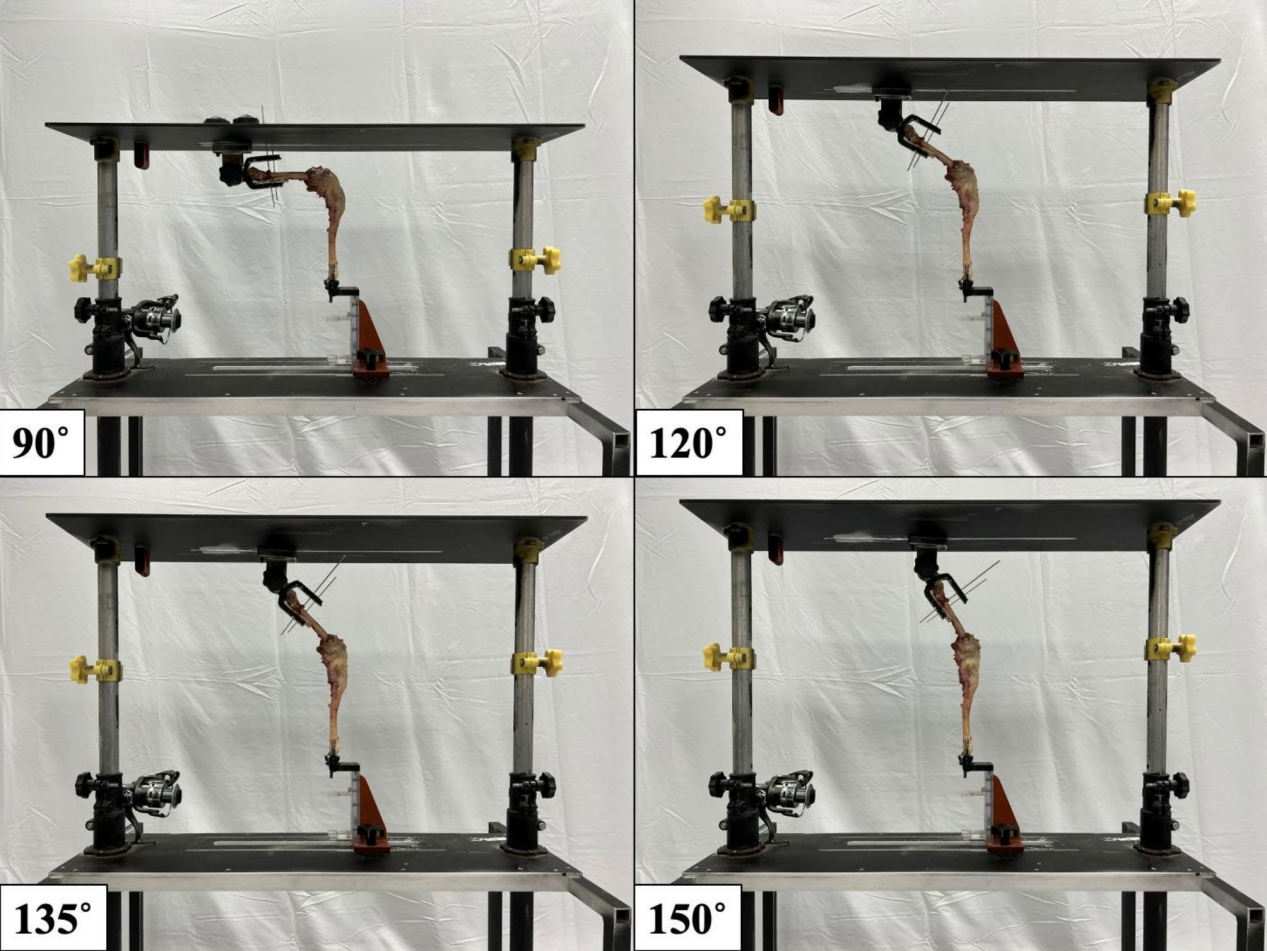
The setup of cadaveric limbs on the custom-made testing platform at four stifle opening angles (i.e., 90°, 120°, 135°, and 150°)

The CD stability test was executed by sequentially applying 20-N resultant caudal and cranial forces provided by hanging weights through nylon lines tied at a bone tunnel caudal to the distal tibial crest. As a result, the applied loadings were steadily maintained to ensure stable tibial caudal and cranial displacements. The height of the nylon line tied with weights was also adjusted on the vertical columns to ensure that the force direction was vertical to the tibial long axis (Fig. 5A). The tibial IR test was executed by quantifying the maximal tibial IR angle under the equivalent torque of 0.6 Nm measured with a torsionmeter (HT-100, Algol Instrument Co., Ltd., Taoyuan City, Taiwan). The CD and tibial IR tests were carried out 3 times at each of 4 stifle opening angles (i.e., 90°, 120°, 135°, and 150°) (Fig. 6). The applied Cr/Cd forces and axial torque were determined in a preliminary study. The 20 N tensile force could yield significant tibial displacement without permanent soft tissue deformation during the cyclic tests and the applied torque of 0.6 Nm could yield an increment of tibial IR angle close to those achieved when sitting and trotting (e.g., 6°-15°) [39].

After tests on the intact CCL stifles were completed, the same procedures were applied to iatrogenic CCL-transected stifles (here referred to as CCLD stifles) and the CCLD stifles repaired with ECS at two different pairs of quasi-isometric points (here referred to as ECS-IP1 and ECS-IP2). During all the abovementioned tests, the marker trajectories along with the skeletal motions were acquired by the motion capture system and managed with a laptop installed with data processing software (Nexus, VICON).

### Preparation of the CCLD and ECS groups

For the preparation of the CCLD groups, limited lateral arthrotomy with a 2-cm incision length was performed by the same surgeon (WRH) to transect the CCL completely with an 11# scalpel blade. The incised joint capsule was subsequently closed with a 3 − 0 polydioxanone suture (PDS II, Ethicon, Raritan, NJ) with a simple continuous suture. The mobility and stability tests were performed again under iatrogenic CCLD status and then repeated after the CCLD stifle was treated with ECS using bone anchors attached to different pairs of quasi-IPs.

**Fig. 7:**
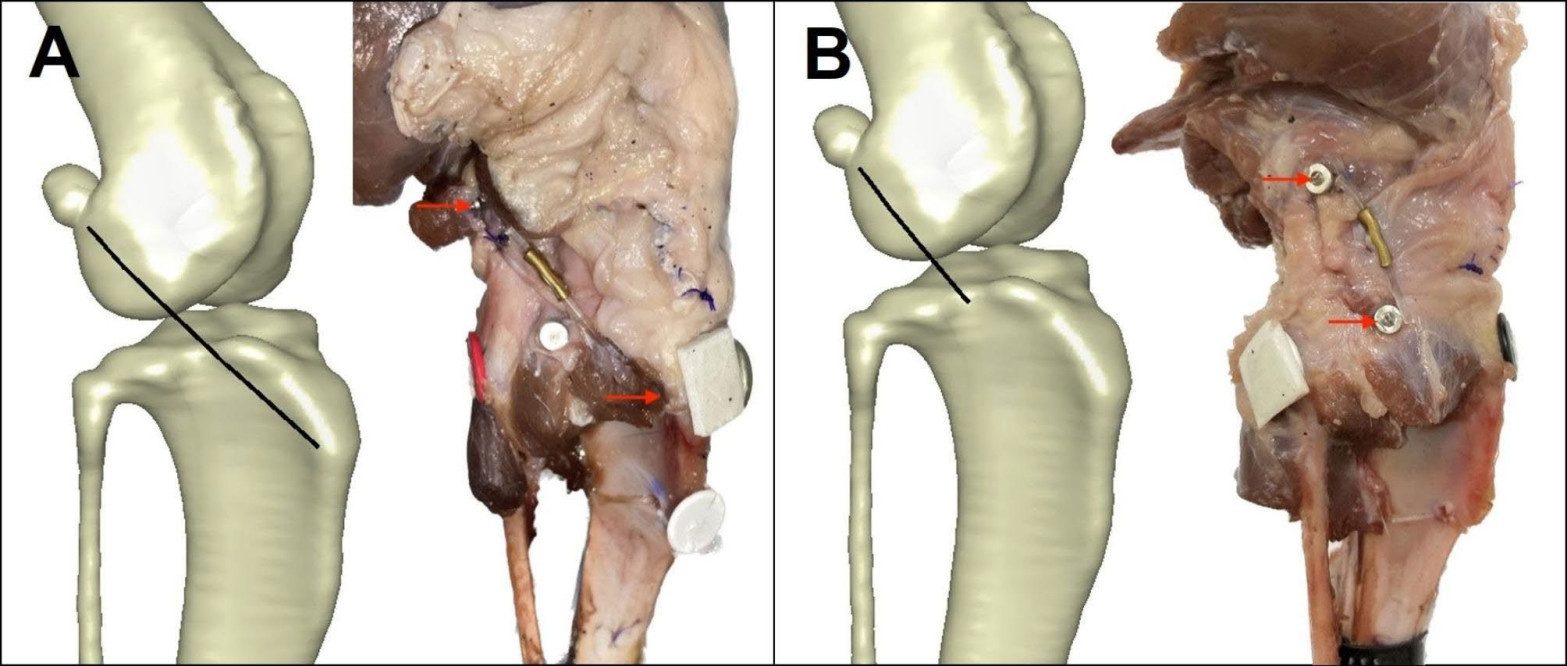
Location of the suture anchor points for the ECS procedure. (**A**) ECS-IP1: the tibia anchor point is located approximately 4–5 mm caudal to the insertion point of the patellar ligament. (**B**) ECS-IP2: the tibial anchor point is located at the tubercle caudal to the long digital extensor groove. The red arrows indicate the suture anchor sites in situ

According to the suggestions in previous studies [12, 20], at the lateral condyle of the femur, the point distal to the lateral femoral fabella (F2) was chosen for the suture attachment site. The site was paired with the point located approximately 4–5 mm caudal to the insertion point of the patellar ligament (equivalent to T1 in Hulse et al [20] and referred to as ECS-IP1 in the present study, Fig. 7A) and the point at the tubercle caudal to the long digital extensor groove (equivalent to T3 in Hulse et al. [20] and referred to as ECS-IP2, Fig. 7B). As a result, the ECS sequentially utilizing the two pairs of quasi-IPs underwent stability and mobility tests.

First, a 3.2 mm pilot hole was predrilled at the lateral femoral condyle in a proximal-anterior direction with a 3.2 mm drill bit (Securos Inc., Fiskdale, MA) to engage the substantive metaphyseal bone. Afterward, a 3.5 mm stainless steel anchor (Securos Inc.) was inserted into the predrilled hole with a hand chuck until the eyelet of the anchor was flush with the bone surface. For tibial anchor placement, steps similar to those for the femoral anchor site were followed for the ECS-IP2 group. However, for the ECS-IP1 group, owing to the thinness of the tibial tuberosity, the bone anchor method was substituted by using a 2.5 mm bone tunnel at the point near the tibial tuberosity to avoid potential instability.

For the ECS-IP1 group, an 80 lb monofilament nylon leader (MNL) line (Securos Inc.) was pulled through the eyelet of the bone anchor at the femoral attachment site and threaded through the bone tunnel. The MNL line was passed through a button and led back to the bone tunnel. Both tips of the leader line were temporarily fixed at the lateral side of the stifle with an 80# titanium nitride-coated stainless steel crimp clamp (Securos Inc.). Two terminals and a Gelpi retractor were used to create tension on the MNL line, and the tension was adjusted until the manual CD test was negative. Preconditioning was executed by creating the passive full range of stifle extension and flexion 20 times. Afterward, the manual CD test was performed once again to ensure negative results. The same operator (WRH) then used a crimper (PowerX crimping device, Securos Inc.) to apply the crimp clamp at a stifle joint angle of 135°. For the ECS-IP2 group, an identical MNL line was directly threaded through the eyelets of the 2 bone anchors and secured with the crimp clamp following the procedures in the ECS-IP1 group.

### Kinematic analysis

The marker data obtained were manually labeled using Nexus software. The anatomical frames of the femur and tibia were determined with anatomical markers following a published report [43]. The marker templates in the respective anatomical frame were used to match the tracking marker locations frame-by-frame as accurately as possible, providing 3D reconstructed skeletal poses [44]. The stifle joint angles were defined as the rotations of the tibia relative to the femur and expressed in F/E, Abd/Add, and IR/ER. The Cr/Cd, proximal/distal (P/D), and lateral/medial (L/M) translations of the stifle were defined as the linear displacements of the stifle joint center with respect to the tibial anatomical frame, in which the stifle joint center was defined as the midpoint of the lateral and medial femoral epicondyles.

For the CCLD and ECS groups, the kinematic deviations relative to the intact CCL in Abd/Add, IR/ER, and stifle joint center translations were quantified as their mean differences in kinematic waveforms throughout stifle F/E. The CTT magnitude was defined as the maximal difference of the stifle joint center positions in the Cr/Cd directions throughout the CD tests, which is equivalent to the maximal tibial Cr/Cd displacement under loadings. The tibial IR angle was defined as the maximal IR angle of the stifle under external torque. The abovementioned kinematics analysis was carried out using a self-developed motion analysis program utilizing MATLAB (R2020a, MathWorks Inc., Natick, MA).

### Statistical analysis

The Shapiro‒Wilk test was used to examine data normality. One-way repeated measure analysis of variance was used to compare kinematic variables among different CCL statuses for data satisfying the normality assumption. Otherwise, the Friedman test was used. In all the above tests, Bonferroni adjusted pairwise t tests were conducted for post hoc analysis, with a significance level *α* = 0.05. The Wilcoxon signed-rank test was applied to examine whether the kinematic deviations were significantly different from 0. The statistical analysis was conducted using SPSS (ver. 26.0, International Business Machines Corp., Armonk, NY).

## Data Availability

The datasets used and/or analysed during the current study are available from the corresponding author on reasonable request.
